# The effect of extended postoperative oral antibiotic prophylaxis on the reinfection risk following two-stage exchange arthroplasty for hip and knee periprosthetic joint infection: a systematic review and meta-analysis

**DOI:** 10.1186/s12891-025-09410-4

**Published:** 2026-01-08

**Authors:** Pei-En Kao, Chih-Wei Hsu, Allen Herng Shouh Hsu, Cheng-Ta Wu, Po-Chun Lin, Feng-Chih Kuo

**Affiliations:** 1 Department of Orthopaedic Surgery, Kaohsiung Chang Gung Memorial Hospital, No. 123, Dapi Road, Niaosong District, Kaohsiung, 833 Taiwan; 2 College of Medicine, Chang Gung University, Kaohsiung, Taiwan; 3 Department of Psychiatry, Kaohsiung Chang Gung Memorial Hospital, Chang Gung University College of Medicine, Kaohsiung, Taiwan; 4 Center for General Education, Cheng Shiu University, Kaohsiung, Taiwan

**Keywords:** Extended postoperative oral antibiotic prophylaxis, Prophylactic antibiotics, Periprosthetic joint infection, Two-stage exchange arthroplasty, Reinfection

## Abstract

**Background:**

This study aimed to conduct a systematic review and meta-analysis to assess the impact of extended postoperative oral antibiotic prophylaxis (EPOAP) on the risk of reinfection following two-stage exchange arthroplasty for hip and knee periprosthetic joint infection (PJI).

**Methods:**

A comprehensive search of PubMed, Embase, and the Cochrane Library was conducted on January 11, 2025. Studies reporting reinfection rates in patients receiving EPOAP after two-stage exchange arthroplasty, compared to those who did not, were included. A random-effects model was used to calculate pooled risk ratios (RRs) and subgroup analyses were performed based on the duration of EPOAP use (> 2 weeks vs. ≤2 weeks) and the joint site (hip or knee).

**Results:**

Five studies, including four retrospective cohort studies and one randomized controlled trial, with a total of 486 knees and 444 hips, were included. The meta-analysis demonstrated that EPOAP significantly reduced the risk of reinfection following two-stage exchange arthroplasty (pooled RR: 0.52; 95% confidence interval [CI]: 0.35, 0.75; *p* = 0.0006). Subgroup analyses yielded similar findings, with significant reductions in reinfection risk for EPOAP duration > 2 weeks (pooled RR: 0.51; 95% CI: 0.34, 0.78; *p* = 0.002), hip arthroplasty (pooled RR: 0.37; 95% CI: 0.20, 0.70; *p* = 0.002), while knee arthroplasty showed a borderline nonsignificant reduction (pooled RR: 0.64; 95% CI: 0.39, 1.04; *p* = 0.07). Only one cohort study reported a decreased risk of reinfection with EPOAP use ≤ 2 weeks. Two studies assessed adverse events related to EPOAP. No complications were observed among 22 patients in one cohort study, whereas 6 of 22 patients (27%) in an RCT experienced mild gastrointestinal or dermatologic reactions that led to treatment discontinuation. Additionally, another cohort study reported that 16 of 24 reinfection cases (67%) involved organisms resistant to EPOAP.

**Conclusions:**

This meta-analysis suggests that EPOAP is associated with a reduced risk of reinfection following two-stage exchange arthroplasty for hip arthroplasty, while further research is warranted for knee cases. This approach may improve patient outcomes and help optimize post-operative antibiotic management.

**Supplementary Information:**

The online version contains supplementary material available at 10.1186/s12891-025-09410-4.

## Background

Total hip and knee replacements are well-established surgical procedures for treating end-stage osteoarthritis, providing sustained functional improvement and enhanced quality of life over the long term [1, 2]. With population aging and the increasing prevalence of osteoarthritis [3], the demand for total hip and knee replacements is rising, with the annual number of procedures projected to reach around two million by 2040 [4]. As the number of procedures increases, the incidence of associated complications, including periprosthetic joint infection (PJI), has also shown an upward trend [5, 6]. PJI is a major contributor to prosthetic failure, accounting for 21.2% of failures in total knee arthroplasty and 19.1% of failures in total hip arthroplasty [7, 8]. Current management strategies for PJI include debridement, antibiotics, and implant retention (DAIR), one- or two-stage exchange arthroplasty, permanent resection arthroplasty, arthrodesis, or amputation [9, 10]. Among these, two-stage exchange arthroplasty is widely regarded as the gold standard for the treatment of chronic PJI [11–13]. However, approximately 10%−15% of patients undergoing two-stage exchange arthroplasty for the hip or knee still experience reinfection [14, 15]. This results in significant burden on both the healthcare system and patients, leading to increased healthcare costs, prolonged hospital stays, and the need for additional surgical interventions [16].

Recently, the use of extended postoperative oral antibiotic prophylaxis (EPOAP) for reinfection prevention after the second stage reimplantation has attracted attention, with several studies emerging but yielding conflicting results. A randomized controlled trial (RCT) demonstrated that EPOAP after two-stage revision total hip or knee arthroplasty significantly reduced the risk of reinfection [17], with some cohort studies showing a similar trend [18, 19]. However, other studies reported no significant impact of EPOAP on reinfection risk, indicating ongoing controversy [20, 21]. Moreover, most of these studies did not evaluate the effects of short-term versus long-term EPOAP use following second-stage reimplantation.

It is hypothesized that EPOAP provides a protective effect against reinfection following the reimplantation. Therefore, this study aimed to systematically review the existing literature on the use of EPOAP after the second stage of two-stage exchange arthroplasty and their impact on reinfection risk.

## Methods

This systematic review and meta-analysis adhered to the Cochrane handbook for systematic reviews of interventions (version 6.5) [22], and Preferred Reporting Items for Systematic Reviews and Meta-Analyses (PRISMA) guidelines [23]. The study protocol was registered in PROSPERO (registration number: CRD42025649154).

### Search strategy

Two investigators (PEK and FCK) systematically searched PubMed, Embase, and the Cochrane Library from database inception to January 11, 2025. The detailed search strategies for each database were as follows: (1) PubMed (“mouth“[MeSH Terms] OR “mouth“[All Fields] OR “oral“[All Fields]) AND (“anti bacterial agents“[MeSH Terms] OR “antibiotic*“[Title/Abstract] OR “bacteriocid*“[Title/Abstract] OR “Antibacterial“[Title/Abstract] OR “anti bacterial“[Title/Abstract] OR “anti bacterial“[Title/Abstract]) AND (“two-stage“[Title/Abstract] OR “two-stage“[Title/Abstract] OR “2-stage“[Title/Abstract] OR “2-stage“[Title/Abstract]) AND (“arthroplasty“[MeSH Terms] OR “revision*“[Title/Abstract] OR “reimplantation*“[Title/Abstract] OR “exchange*“[Title/Abstract] OR “arthroplast*“[Title/Abstract]) AND (“hip“[Text Word] OR “knee“[Text Word] OR “hips“[Text Word] OR “knees“[Text Word]). (2) Embase: (‘mouth’/exp OR ‘mouth’:ti, ab OR ‘oral’:ti, ab) AND (‘antiinfective agent’/exp OR ‘antibiotic*’:ti, ab OR ‘bacteriocid*’:ti, ab OR ‘antibacterial’:ti, ab OR ‘anti-bacterial’:ti, ab OR ‘anti bacterial’:ti, ab) AND (‘two-stage revision’/exp OR ‘two-stage’:ti, ab OR ‘two stage’:ti, ab OR ‘2-stage’:ti, ab OR ‘2 stage’:ti, ab) AND (‘reimplantation*’ OR ‘exchange*’ OR ‘arthroplast*’ OR ‘revision*’ OR ‘arthropathy’/exp) AND (‘hip’ OR ‘hips’ OR ‘knee’ OR ‘knees’). (3) Cochrane Library: ((MeSH descriptor: [Mouth] explode all trees) OR (mouth) OR (oral)) AND ((MeSH descriptor: [Anti-Bacterial Agents] explode all trees) OR (antibiotic*):ti, ab, kw OR (bacteriocid*):ti, ab, kw OR (Antibacterial): ti, ab, kw OR (Anti-Bacterial): ti, ab, kw OR (Anti Bacterial): ti, ab, kw) AND ((two-stage): ti, ab, kw OR (two stage): ti, ab, kw OR (2 stage): ti, ab, kw) AND ((MeSH descriptor: [Arthroplasty] explode all trees) OR (revision*):ti, ab, kw OR (reimplantation*):ti, ab, kw OR (exchange*):ti, ab, kw OR (arthroplast*):ti, ab, kw) AND ((hip) OR (hips) OR (knee) OR (knees)). No limitations were imposed on study design, publication date, and language; therefore, gray literature and conference abstracts were not excluded during the search process. Furthermore, a snowballing approach was employed, involving manual screening of bibliographic references and citations of included studies and relevant reviews to identify additional eligible articles.

### Study selection

The present meta-analysis was conducted based on the PICO framework (Population, Intervention or exposure, Comparison, and Outcome) as follows: P: human participants who underwent two-stage exchange arthroplasty for hip or knee PJI; I: EPOAP; C: no EPOAP; and O: reinfection rate.

The inclusion criteria for this meta-analysis were as follows: (1) studies involving human participants who underwent two-stage exchange arthroplasty for hip or knee PJI; (2) studies assessing the association between the risk of reinfection and EPOAP after second-stage reimplantation; (3) a control group consisting of patients who did not receive EPOAP following second-stage reimplantation; (4) studies reporting data on the incidence of reinfection after second-stage reimplantation; and (5) study designs comprising cohort studies as well as randomized or non-randomized controlled trials. The control group receiving a short duration of oral or intravenous antibiotics, limited to no more than 3 days, after undergoing second-stage reimplantation, was acceptable, as it aligned with the protocol of the control group in two previous cohort studies [18, 20].

The exclusion criteria were as follows: (1) study designs including conference abstracts, case-control studies, cross-sectional studies, review articles, editorials, letters to the editor, and studies without available full texts; (2) studies in which EPOAP were only administered between the first and second stages of exchange arthroplasty; and (3) studies where the route of antibiotic administration was parenteral. The type and duration of EPOAP, as well as the age of population were not limited. If two or more articles originated from the same clinical trial, the more recent one was retained due to its more complete data.

Two investigators (PEK and FCK) independently screened the eligibility of articles based on the titles and abstracts. The remaining studies were retrieved through full-text review according to the inclusion and exclusion criteria. Disagreements were addressed through consulting the third reviewer (CWH).

### Quality assessment

The risk of bias in the included cohort studies or non-randomized controlled trials was assessed using the Methodological Index for Non-randomized Studies (MINORS) scoring system [24]. Each of the 12 items was scored from 0 to 2, where 0 indicates not reported, 1 indicates reported but inadequate, and 2 indicates reported and adequate. The maximum possible score was 16 for non-comparative and 24 for comparative studies. We applied Version 2 of the Cochrane risk of bias tool for randomized trials (RoB 2.0) for evaluating the risk of bias of the included randomized controlled trials (RCTs) [25]. This tool focused on five domains (randomization process, adherence to the intervention, missing outcome data, outcome measurement, and selective reporting), and then provided an overall risk of bias judgment. Furthermore, the level of evidence for each included study was assessed according to the 2011 Oxford Centre for Evidence-Based Medicine (OCEBM) classification system [26]. Within this framework, Level 2 evidence refers to randomized controlled trials or well-designed observational studies demonstrating a large effect, whereas Level 3 evidence pertains to non-randomized controlled cohort or follow-up studies. The assigned level could be downgraded in the presence of methodological limitations such as risk of bias, imprecision, indirectness, inconsistency across studies, or a very small effect size, and upgraded if a large or very large effect size was observed. Two investigators (PEK and FCK) independently assessed the risk of bias and the levels of evidence of the included studies. Any disagreements were resolved by consulting the third reviewer (CWH).

### Outcomes assessment

The primary outcome of interest was the risk of reinfection in patients with EPOAP, compared to those without, after the second-stage reimplantation. The secondary outcomes included adverse events or complications associated with EPOAP, the number of patients who discontinued EPOAP due to these adverse events, and the proportion of reinfection cases caused by the same pathogen with same sensitivity identified at the first stage.

### Data extraction

Two investigators (PEK and FCK) independently extracted data from the selected studies using a standardized form created in Microsoft Excel. Extracted information included study design, first author, country, year of publication, age, gender, follow-up duration, number of patients and sample sizes for the study and control groups, definition of incident reinfection, the incidence of reinfection, covariates adjusted for, type and duration of EPOAP following the second-stage reimplantation, post-operative treatment of the control group, the joint site of arthroplasty (hip or knee), risk estimates (odds ratio, risk ratio (RR), incidence rate ratio, or hazard ratio (HR)) along with their 95% confidence intervals (CIs), the adverse events or complications related to EPOAP, the number of patients who were unable to complete EPOAP due to intolerable side effects, and the incidence of reinfection cases caused by the same pathogen with same sensitivity.

### Data analyses and software

Bivariate meta-analyses were conducted to estimate the risk of incident reinfection following second-stage reimplantation with pooled RRs with 95% CIs by using the Mantel-Haenszel (M-H) method with random-effects models [27]. Subgroup analyses were performed based on the study design (RCT or cohort study), the duration of EPOAP (> 2 weeks or ≤ 2 weeks) and the joint site of arthroplasty (hip or knee). The classification of EPOAP duration into > 2 weeks or ≤ 2 weeks was adopted in accordance with previous cohort study [19]. A sensitivity analysis was performed, restricted to studies that applied the Musculoskeletal Infection Society (MSIS) criteria for the diagnosis of reinfection, since these criteria are widely adopted in clinical practice [28]. Meta-regression analysis was performed to investigate whether the reinfection risk observed in the control groups, who did not receive EPOAP and thus represent the baseline risk, was associated with the treatment effect of EPOAP. Also, the proportion of reinfection cases caused by the same pathogen with same sensitivity was expressed as pooled RRs with 95% CIs. To assess publication bias, a funnel plot was generated and visually examined when more than 10 studies were included in the meta-analysis [29].

All statistical analyses were performed using Review Manager (RevMan) 5.4 software (Cochrane Collaboration), except for the meta-regression analysis, which was conducted in R version 4.5.1 using RStudio. The degree of heterogeneity across the included studies was assessed using the I-squared (I²) statistic. I² values below 40% was considered low heterogeneity, values between 40% and 75% suggesting moderate heterogeneity, and values exceeding 75% reflected substantial heterogeneity [30]. A p-value of less than 0.05 was considered statistically significant.

During the preparation of this work, the authors used OpenAI’s Chat Generative Pre-Trained Transformer (ChatGPT) (version GPT-4o and GPT-4o mini) to enhance the clarity and readability of the English text. It was not involved in data analysis, interpretation, or the formulation of conclusions. After using this tool, the authors reviewed and edited the content as needed and take full responsibility for the content of the publication.

## Results

### Search results

**Fig. 1:**
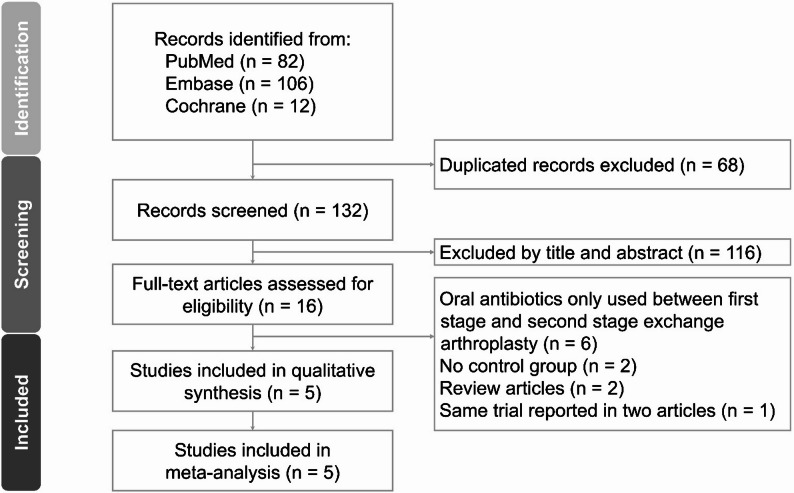
Preferred Reporting Items for Systematic Reviews and Meta-analyses (PRISMA) flowchart

Initially, 200 studies were retrieved from three databases. After removing duplicates and screening the titles and abstracts, 16 studies were identified for further evaluation. After reviewing the full text, 11 studies were excluded, with the reasons for exclusion provided in Additional file 1. Four cohort studies [18–21] and one RCT [17] were included in the systematic review and meta-analysis. The study selection process was illustrated in the PRISMA 2020 flow diagram (Fig. 1), and the characteristics of the included studies are summarized in Table 1. A summary of the data extraction from the included studies is provided in Additional file 2.

**Table 1:** Characteristics of included studies

Author/Year	Study design (Country)	Age (years) and gender (male/female) (Study group vs. control group)	Study group No. of joints	Type of EPOAP	Duration of EPOAP (Range)	Control group No. of joints	Postoperative treatment (Mean; Range)	The definition of reinfection	Follow-up duration (Study group vs. control group)
Zywiel 2011 [18]	RCS (USA)	Mean (range): 63 (40 to 89) in the both groups Gender: NR	28 knees	TMP/SMX, linezolid, ciprofloxacin, cephalexin, and doxycycline	(28 to 43 days)	38 knees	Parenteral abx (1.3 days; 1 to 3 days)	Leone and Hanssen criteria	Minimum 12 mo
Johnson 2013 [20]	RCS (USA)	Mean (range): 58 (26 to 87) vs. 57 (19 to 89) Gender: 12/10 vs. 17/27	23 hips	NR	Mean: 36 days (14 days to lifelong)	44 hips	Parenteral abx (1.3 days; 1 to 3 days)	Leone and Hanssen criteria	Mean (range): 45 mo (24 mo to 105 mo)
Yang 2020 [17]	RCT (USA)	Mean (SD): 62.9 (10.8) vs. 63.3 (10.5) Gender: 45/27 vs. 42/28	31 hips 41 knees	NR	3 mo	32 hips 38 knees	No additional abx	MSIS criteria	Mean (SD): 3.43 mo (1.43) vs. 3.21 mo (1.30)
Kelly 2022 [21]	RCS (USA)	Mean (SD): 61.5(11.1) vs. 61.0 (11.1) Gender: 76/82 vs. 22/31	75 hips 83 knees	Doxycycline, beta-lactams, quinolones, TMP/SMX, minocycline, ciprofloxacin, and cefadroxil	> 12 weeks in 124 pts 4 to 12 weeks in 27 pts 2 to 4 weeks in 7 pts	29 hips 24 knees	No additional abx	MSIS criteria	Mean (SD): 2.2 mo (1.7) (minimum 1 year)
Ryan 2023 [19]	RCS (USA)	Mean (range); gender: Group A: 65 (59 to 73); 161/105 Group B: 66 (61 to 73); 41/35 Controls: 68 (60 to 75); 55/47	Group A: 133 hips + 133 knees Group B: 34 hips + 42 knees	NR	Group A: ≤ 2 weeks (3 to 14 days) Group B: > 2 weeks (15 days to 6 mo)	43 hips 59 knees	No additional abx or intravenous abx < 48 h	MSIS criteria	Median (range): 2.2 years (3 days to 10.5 years)

Abx: antibiotics; EPOAP: extended postoperative oral antibiotic prophylaxis; mo: months; MSIS criteria: Musculoskeletal Infection Society Criteria; No.: Number; NR: not reported; pts: patients; RCS: retrospective cohort study; RCT: randomized controlled trial; SD: standard deviation; TMP/SMX: trimethoprim/sulfamethoxazole

### Quality assessment and publication bias

The risk of bias for the included studies is outlined in Table 2. Four cohort studies [18–21] achieved MINORS scores ranging from 16 to 19 out of a maximum of 24 points for comparative studies and were classified as level 3 evidence. The overall risk of bias for the RCT was classified as having some concerns, as both participants and investigators were not blinded; this study was categorized as level 2 evidence [17]. Since no more than ten studies were included in the meta-analysis, a funnel plot to assess publication bias was not conducted.

**Table 2:** Risk of bias of included studies using Cochrane risk of bias 2 tool for randomized controlled trials and methodological index for Non-Randomized studies for cohort studies

Author/Year		MINORS score (A maximum score = 24 for comparative studies)						Levels of Evidence
Zywiel 2011 [11]		16/24						3
Johnson 2013 [20]		18/24						3
Kelly 2022 [21]		19/24						3
Ryan 2023 [19]		19/24						3

Author/Year	Randomization process		Intervention adherence	Missing outcome data	Outcome measurement	Selective reporting	Overal RoB	Levels of Evidence
Yang 2020 [17]	L		L	L	S	L	S	2

Stars (★) were assigned in accordance with the Newcastle-Ottawa Quality Assessment Form for Cohort Studies. L: low risk of bias; RoB: risk of bias; MINORS: Methodological Index for Non-Randomized Studies; S: some concerns

### Risk of reinfection in patients receiving EPOAP following second-stage reimplantation

**Fig. 2:**
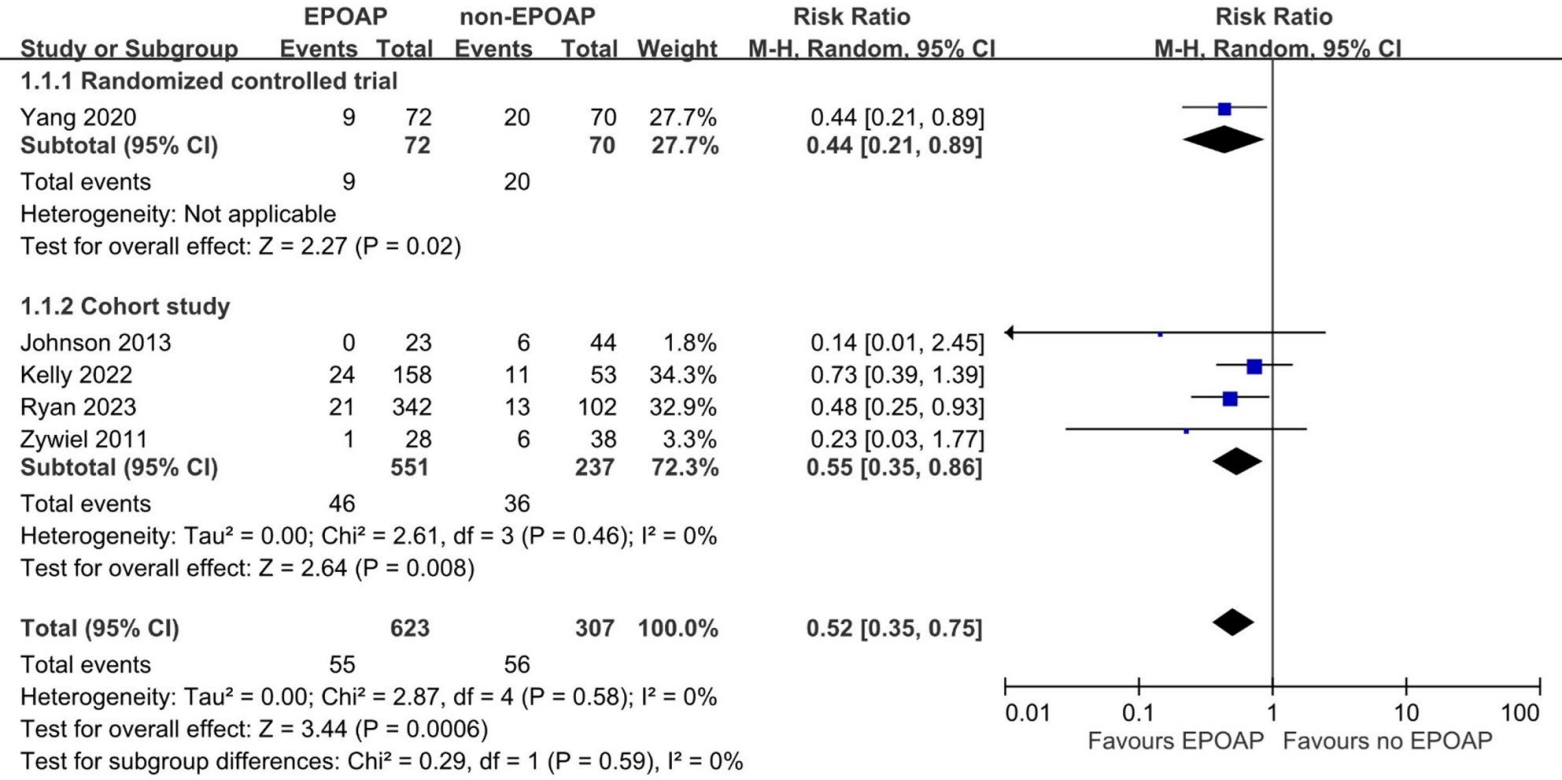
Forest plot illustrating the association between extended postoperative oral antibiotic prophylaxis (EPOAP) and the risk of reinfection following two-stage exchange arthroplasty for hip or knee. Subgroup analyses were conducted based on study design, including randomized controlled trials and cohort studies

Five studies, including a total of 444 hips and 486 knees, were conducted to assess the risk of reinfection in patients receiving EPOAP following second-stage reimplantation [17–21]. The absence of reinfection at the time of reimplantation was reported in these studies. As shown in Fig. 2, the meta-analysis indicated that EPOAP significantly decreased the risk of reinfection following second-stage reimplantation (pooled RR: 0.52; 95% confidence interval [CI]: 0.35, 0.75; *p* = 0.0006), with low heterogeneity (I² = 0%) across the five studies.

**Fig. 3:**
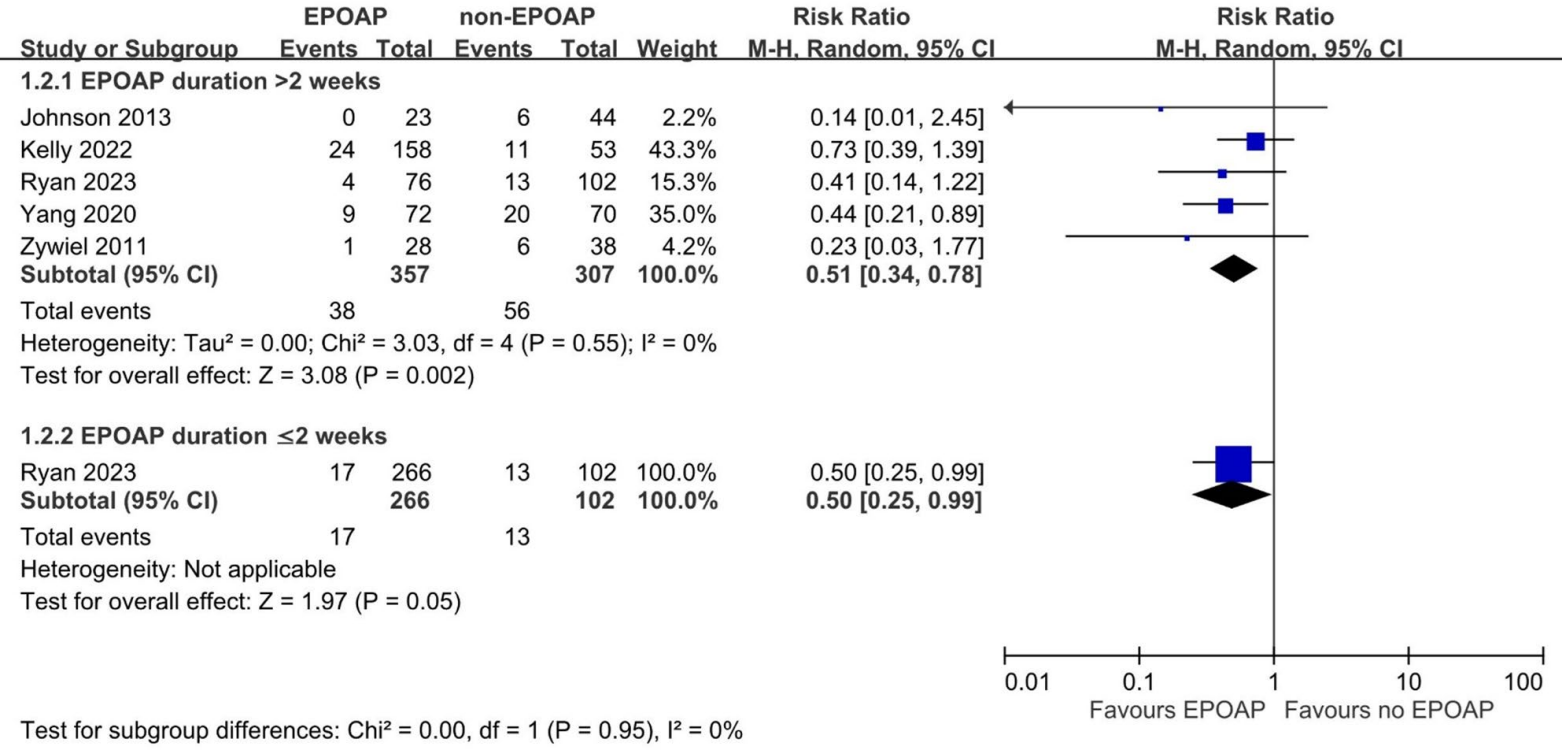
Forest plot of the association between extended postoperative oral antibiotic prophylaxis (EPOAP) and the risk of reinfection following two-stage exchange arthroplasty for hip or knee. Subgroup analyses were performed based on the duration of oral antibiotic use, categorized as > 2 weeks or ≤ 2 weeks

**Fig. 4:**
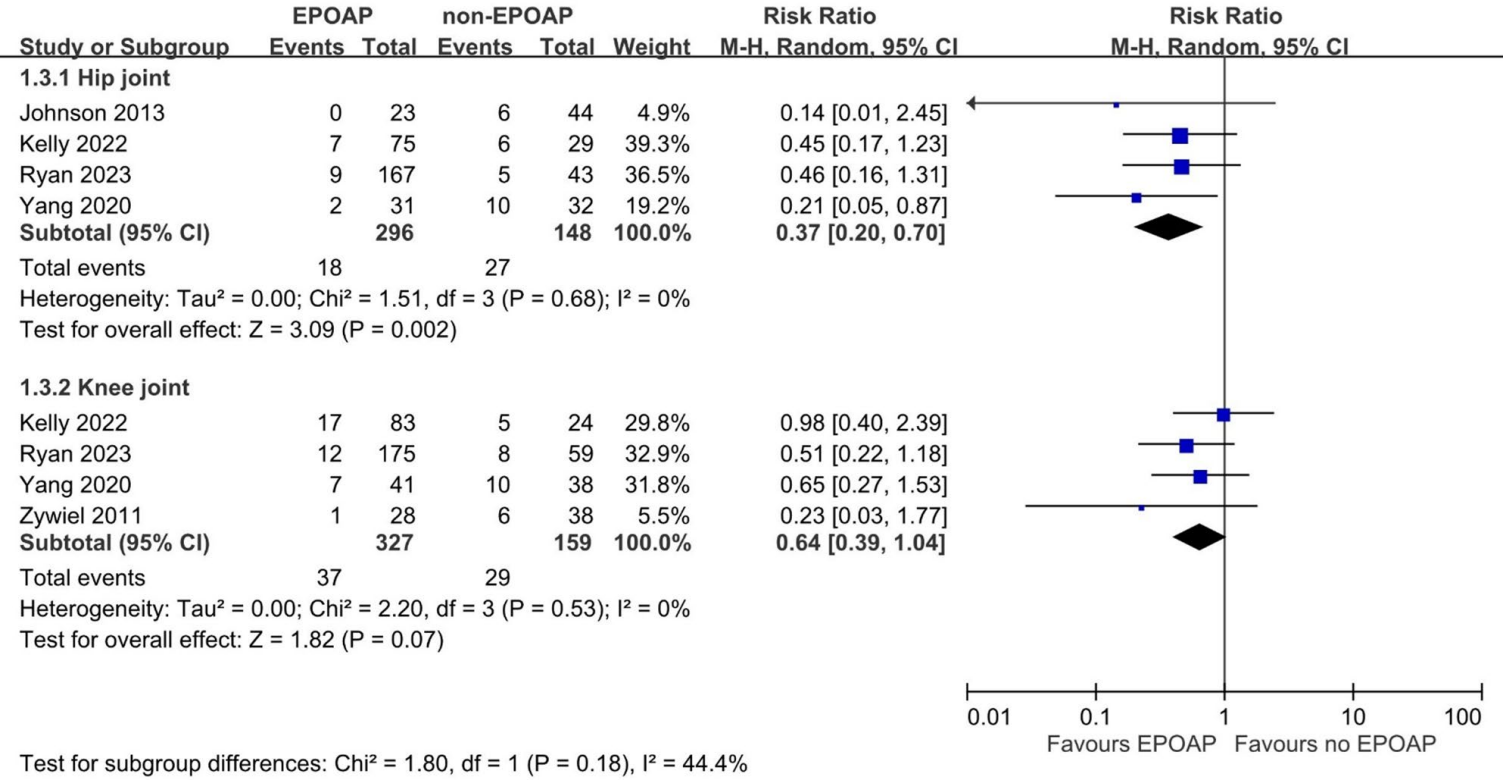
Forest plot depicting the association between extended postoperative oral antibiotic prophylaxis (EPOAP) and the risk of reinfection following two-stage exchange arthroplasty for hip or knee. Subgroup analyses were according to the joint site, including hip joint or knee joint

**Fig. 5:**
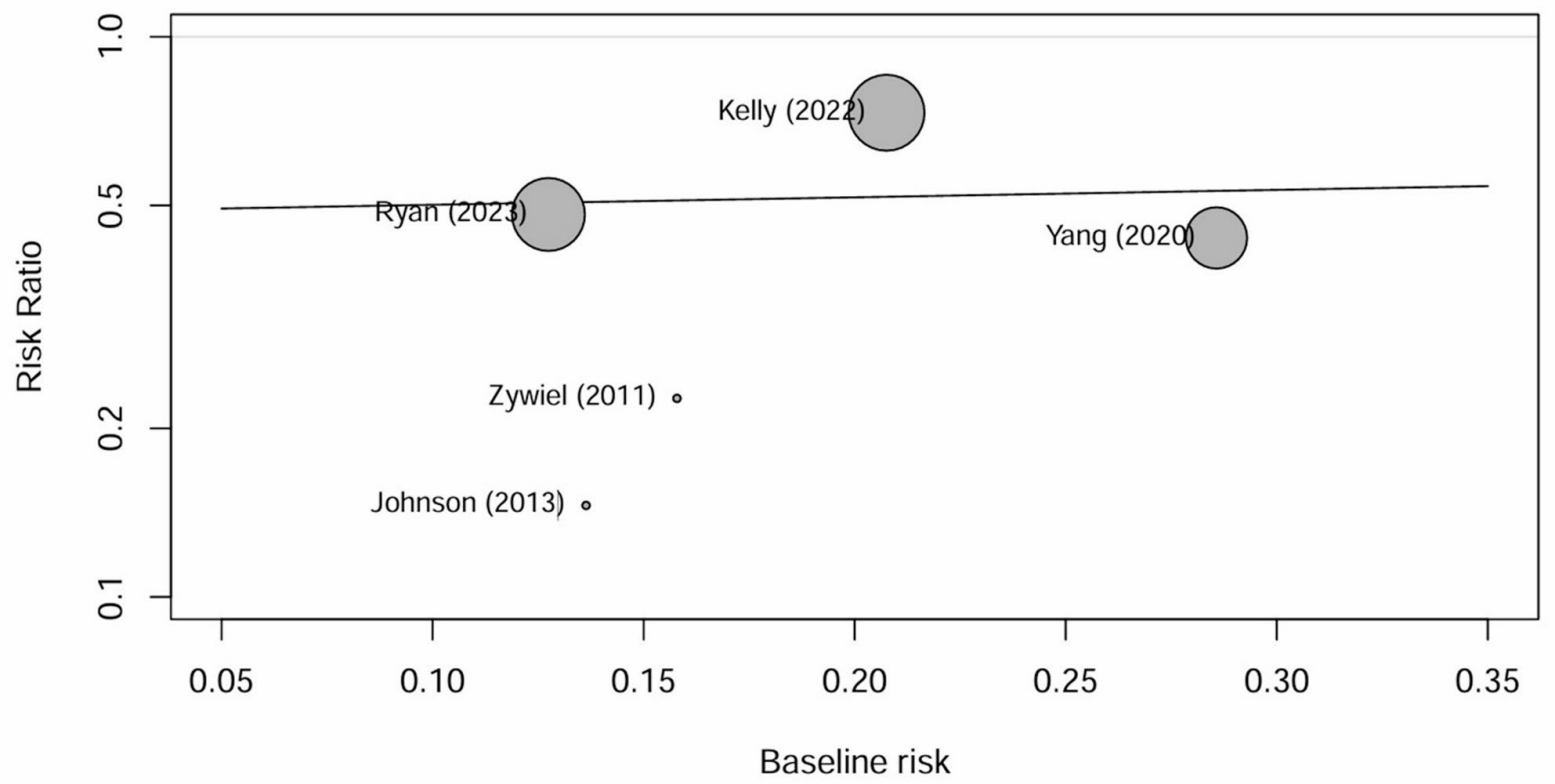
Meta-regression assessing the relationship between reinfection risk in the control groups (baseline risk) and the treatment effect of extended postoperative oral antibiotic prophylaxis (risk ratio). The coefficient (β = 0.307) is expressed on the natural logarithm (ln) scale, with a *p*-value of 0.92

Subgroup analyses, illustrated in Figs. 2 and 3, and 4, found similar results, including for four cohort studies (pooled RR: 0.55; 95% CI: 0.35, 0.86; *p* = 0.008; I² = 0%), EPOAP duration > 2 weeks (pooled RR: 0.51; 95% CI: 0.34, 0.78; *p* = 0.002; I² = 0%), and hip joint (pooled RR: 0.37; 95% CI: 0.20, 0.70; *p* = 0.002; I² = 0%). A nonsignificant result was found for knee joint (pooled RR: 0.64; 95% CI: 0.39, 1.04; *p* = 0.07; I² = 0%). A sensitivity analysis restricted to three cohort studies [17, 19, 21] using the MSIS criteria demonstrated a statistically significant reduction in reinfection risk (pooled RR: 0.54; 95% CI: 0.37, 0.80; *p* = 0.002; I² = 0%). In Fig. 5, the meta-regression coefficient for reinfection risk in the control groups was 0.307 on the natural logarithm (ln) scale, with a p value of 0.92, indicating no significant association between baseline risk and treatment effect.

**Fig. 6:**
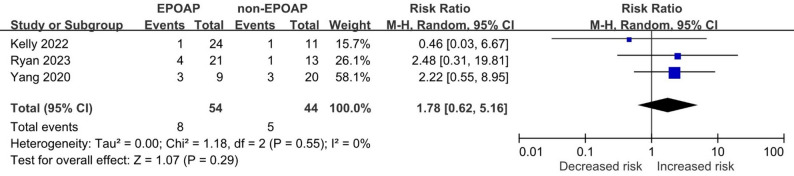
Forest plot illustrating the association between extended postoperative oral antibiotic prophylaxis (EPOAP) and the proportion of reinfection cases caused by the same pathogen with identical sensitivity

Two cohort studies and one RCT reported the proportion of reinfection cases caused by the same pathogen with same sensitivity [17, 19, 21]. No significant difference was found in EPOAP (pooled RR: 1.78; 95% CI: 0.62, 5.16; *p* = 0.29; I² = 0%) in Fig. 6.

### Adverse events or complications related to EPOAP

Two studies assessed adverse events or complications associated with EPOAP [17, 20]. In a cohort study, no complications were observed in 22 patients (23 hips) [20]. In contrast, the RCT reported six patients receiving doxycycline experiencing adverse events, including diarrhea (3 cases), nausea (2 cases), vomiting (1 case), pruritus (1 case), and rash (3 cases) [17]. Additionally, one patient receiving ciprofloxacin experienced abdominal cramps. All six patients discontinued EPOAP due to these adverse events.

## Discussion

### Main findings and clinical relevance

The present systematic review and meta-analysis revealed that EPOAP is associated with a decreased risk of reinfection following the second-stage reimplantation for PJI of the hip or knee. Subgroup analyses yielded consistent findings across cohort studies, EPOAP durations exceeding two weeks, and hip arthroplasty. However, results for knee arthroplasty were not statistically significant and remain inconclusive. Reinfection after two-stage exchange arthroplasty is a devastating complication, often resulting in repeated surgeries, prolonged hospitalization, increased mortality, and considerable healthcare costs [31–33]. For the patients who develop recurrent infection after a prior two-stage exchange frequently require further surgical interventions, which are associated with high failure rates—reported as approximately 40% to 60%—including persistent infection, the need for subsequent revision procedures, or PJI-related mortality [31]. Therefore, strategies that effectively reduce reinfection, such as EPOAP, may have significant clinical value in minimizing the likelihood of two-stage exchange failure and improving patient outcomes.

### Comparison with literature

Beyond the two-stage exchange setting, numerous studies in primary and aseptic revision total hip or knee arthroplasty have also evaluated EPOAP for PJI prevention. A recent meta-analysis including 19,153 patients reported that EPOAP was associated with a reduced risk of PJI after primary total hip arthroplasty, whereas a nonsignificant but downward trend was observed after primary total knee arthroplasty [34]. In aseptic revision procedures, the protective effect of EPOAP was significant for revision total knee arthroplasty but not for revision total hip arthroplasty, although a similar decreasing trend was observed [34]. These findings are generally aligned with our results, indicating a consistent trend toward infection risk reduction with EPOAP across different surgical contexts. However, it remains uncertain whether EPOAP provides a clinically meaningful protective benefit in primary total knee arthroplasty, revision total hip arthroplasty, or two-stage exchange procedures involving the knee. Further studies are warranted to clarify the potential benefit of EPOAP in these specific patient populations.

### Regimen considerations

The selection of EPOAP was mainly tailored to the organism identified through culture at the first stage [17–21]. Most of reinfection incidence was caused by the new organism, regardless of whether the patient received antibiotics. In these three studies, 46 of 54 (85.2%) reinfection events in EPOAP-treated patients and 39 of 44 (88.6%) in untreated patients were caused by either different pathogens or the same pathogens with different sensitivities from those cultured at the initial stage [17, 19, 21]. Hence, the mechanism of EPOAP in inhibiting reinfection may be explained by prophylactically inhibiting the growth of new pathogens. The use of broad-spectrum antibiotics may offer superior efficacy in preventing reinfection by addressing these new pathogens, and further RCT are warranted to confirm this approach.

### Duration of EPOAP

The optimal duration of EPOAP remains unclear. In the included studies, EPOAP have been administered for at least two weeks [17–21], with only one cohort study showing borderline significance for EPOAP used for ≤ 2 weeks [19]. Two cohort studies, involving a total of 133 patients, reported that a mean duration of approximately one month of EPOAP showed a trend toward a lower risk of reinfection, although the difference was not significant, possibly due to small sample size [18, 20]. Another cohort study also observed a similar trend, and 124 out of 158 patients in the EPOAP-treated group received more than 12 weeks of EPOAP [21]. Additionally, the cohort study found that 266 patients treated with EPOAP for no more than two weeks had a lower risk of reinfection, while 76 patients treated for more than two weeks showed no significant difference, likely due to the small sample size [19]. The RCT that a three-month course of EPOAP significantly reduced the risk of reinfection [17]. Therefore, the current evidence may suggest that the use of EPOAP for a duration of approximately one to three months could be beneficial; however, this observation should be regarded as hypothesis-generating rather than conclusive. Notably, the selection and duration of EPOAP should be tailored and adjusted based on the patient’s clinical condition, including clinical manifestations, laboratory test results, underlying diseases, causative pathogens, and the risk of adverse events. Further studies are warranted to determine whether a short course of EPOAP is non-inferior to a long course in preventing reinfection. Importantly, subgroup analysis in total knee arthroplasty revealed no significant difference. Consequently, the available evidence for total knee arthroplasty is inconclusive, underscoring the necessity for RCTs focused specifically on this patient group.

### Safety and adverse events

Regarding the safety profile, the cohort study found that 24 out of 158 EPOAP-treated patients developed reinfection, and 16 out of them had positive cultures with organisms resistant to the EPOAP [21]. In the RCT, only five out of 72 patients discontinued the three-month EPOAP due to intolerable adverse events [17]. Clinicians should be aware of the potential for drug resistance and adverse events when managing reinfection with prolonged EPOAP.

### Strengths and limitations

The present study had several strengths. First, to the best of our knowledge, this was the first systematic review and meta-analysis to demonstrate the efficacy of EPOAP in preventing incident reinfection following two-stage exchange arthroplasty. Second, subgroup analyses showed consistent results, including in cohort study, and hip joints, with a similar trend observed in the knee joint. That said, there were some limitations. First, most of the included studies were retrospective cohort studies. Due to the nature of this study design, confounding bias was inevitable. The data we extracted was before any adjustments. However, EPOAP were more likely to be prescribed to patients with higher infection risk, more comorbidities, or those more susceptible to poor prognosis. Therefore, this confounding by indication might have underestimated the positive effect of EPOAP, and the conclusion is unlikely to change. Second, the duration and type of EPOAP varied widely among the studies, which could introduce heterogeneity in the results and limit the ability to draw definitive conclusions regarding the optimal regimen and duration. This heterogeneity should be considered when interpreting the pooled results. However, low heterogeneity was detected in the meta-analyses. The type and duration of EPOAP should be based on the clinical setting, including the organism identified in initial cultures, its sensitivity to specific antibiotics, and the patient’s underlying conditions [35]. Third, the definition of reinfection following reimplantation differed between studies, contributing to clinical heterogeneity. Two studies using the Leone and Hanssen criteria accounted for only 5.1% of the weight in the meta-analysis [18, 20]. A sensitivity analysis was therefore conducted, only including studies [17, 19, 21] using the MSIS criteria, which are more widely adopted in current clinical practice [28]. This analysis showed results similar to the main meta-analysis. Although the limited number of studies warrants cautious interpretation, these results nonetheless provide clinically relevant evidence supporting the potential of EPOAP to reduce recurrence and improve implant survival after two-stage reimplantation. Such findings highlight the role of EPOAP as a practical adjunct to optimize postoperative infection control in patients at high risk of reinfection. Further well-designed prospective and randomized studies are warranted to confirm and refine these observations. Fourth, with only five studies included, all subgroup and meta-regression analyses in our study may be underpowered and susceptible to spurious findings. Therefore, readers should interpret the results of the subgroup and meta-regression analyses with caution and avoid overgeneralization.

## Conclusions

Our findings provide valuable clinical guidance by supporting the use of EPOAP to reduce the risk of reinfection following second-stage reimplantation for hip arthroplasty. While results for total knee arthroplasty showed no significant difference, further research is warranted in this population. This approach can help improve patient outcomes by minimizing the incidence of reinfection and may inform clinical decisions on post-operative care.

## Supplementary Information

Supplementary Material 1.

Supplementary Material 2.

## Data Availability

All data used in this study are obtained from previously published literature and are fully cited.

## Abbreviations

PJI: Periprosthetic Joint Infection
EPOAP: extended postoperative oral antibiotic prophylaxis
RCT: Randomized Controlled Trial
DAIR: Debridement, Antibiotics, and Implant Retention
MINORS: Methodological Index for Non-randomized Studies
RoB 2.0: Risk of Bias 2.0 (Cochrane risk of bias tool for randomized trials)
PICO: Population, Intervention, Comparison, Outcome
RR: Risk Ratio
CI: Confidence Interval
PRISMA: Preferred Reporting Items for Systematic Reviews and Meta-Analyses
RevMan: Review Manager
ChatGPT: Chat Generative Pre-Trained Transformer
