# Supplementary material for: The effect of extended postoperative oral antibiotic prophylaxis on the reinfection risk following two-stage exchange arthroplasty for hip and knee periprosthetic joint infection: a systematic review and meta-analysis
Source: BMC Musculoskelet Disord. 2026 Jan 8;27:101. doi: 10.1186/s12891-025-09410-4 (PMC12870892; doi:10.1186/s12891-025-09410-4)
Supplement: Supplementary file 1 — Supplementary Material 1. [file 12891_2025_9410_MOESM1_ESM.docx]

**Additional file 1**

**Additional file 1.** Detailed reasons for study exclusion.

| Citations | Reasons |
| --- | --- |
| L Bernard, C Arvieux, B Brunschweiler, et al. Antibiotic Therapy for 6 or 12 Weeks for Prosthetic Joint Infection. N Engl J Med 2021;384:1991-2001. | Intravenous or oral antibiotics were prescribed between first stage and second stage exchange arthroplasty, comparing 6 versus 12 weeks of treatment. |
| ES Darley, GC Bannister, AW Blom, AP Macgowan, SK Jacobson and W Alfouzan. Role of early intravenous to oral antibiotic switch therapy in the management of prosthetic hip infection treated with one- or two-stage replacement. *J Antimicrob Chemother* 2011;66:2405-8. | Oral antibiotics were prescribed between first stage and second stage exchange arthroplasty, with the intervention involving an early switch to oral antibiotics. |
| MB Siqueira, A Saleh, AK Klika, et al. Chronic Suppression of Periprosthetic Joint Infections with Oral Antibiotics Increases Infection-Free Survivorship. *J Bone Joint Surg Am* 2015;97:1220-32. | Oral antibiotics were prescribed between first stage and second stage exchange arthroplasty |
| T Ascione, P Pagliano, G Balato, M Mariconda, R Rotondo and S Esposito. Oral Therapy, Microbiological Findings, and Comorbidity Influence the Outcome of Prosthetic Joint Infections Undergoing 2-Stage Exchange. *J Arthroplasty* 2017;32:2239-2243. | Oral antibiotics were prescribed between first stage and second stage exchange arthroplasty |
| E Senneville, A Dinh, T Ferry, E Beltrand, N Blondiaux and O Robineau. Tolerance of Prolonged Oral Tedizolid for Prosthetic Joint Infections: Results of a Multicentre Prospective Study. *Antibiotics (Basel)* 2020;10. | The timing of oral antibiotics in two-stage exchange arthroplasty was not mentioned, and no control group was included. |
| P Reinecke, P Morovic, M Niemann, et al. Adverse Events Associated with Prolonged Antibiotic Therapy for Periprosthetic Joint Infections-A Prospective Study with a Special Focus on Rifampin. *Antibiotics (Basel)* 2023;12. | The timing of oral antibiotics in two-stage exchange arthroplasty was not mentioned, and no control group was included. |
| DA Hoad-Reddick, CR Evans, P Norman and I Stockley. Is there a role for extended antibiotic therapy in a two-stage revision of the infected knee arthroplasty? *J Bone Joint Surg Br* 2005;87:171-4. | Oral antibiotics were prescribed between first stage and second stage exchange arthroplasty |
| R Pushkin, MD Iglesias-Ussel, K Keedy, et al. A Randomized Study Evaluating Oral Fusidic Acid (CEM-102) in Combination With Oral Rifampin Compared With Standard-of-Care Antibiotics for Treatment of Prosthetic Joint Infections: A Newly Identified Drug-Drug Interaction. *Clin Infect Dis* 2016;63:1599-1604. | Oral fusidic acid and rifampin were prescribed between first stage and second stage exchange arthroplasty, comparing oral fusidic acid and rifampin to intravenous antibiotics. |
| JL Tidd, I Pasqualini, K McConaghy, et al. The Use of Oral Antibiotics After Total Joint Arthroplasty: A Critical Analysis Review. *JBJS Rev* 2023;11. | Review article |
| S Minhas, R Odono, K Collins, J Vigdorchik and R Schwarzkopf. The Role and Timing of Treatment Strategies During Two-Stage Revision for Periprosthetic Joint Infections. *Bull Hosp Jt Dis (2013)* 2017;75:246-247. | Review article |
| JM Frank, E Kayupov, M Moric, et al. The Mark Coventry, MD, Award: Oral Antibiotics Reduce Reinfection After Two-Stage Exchange: A Multicenter, Randomized Controlled Trial. *Clin Orthop Relat Res* 2017;475:56-61. | This study and the study by Yang et al.* originated from the same trial registered on ClinicalTrials.gov (NCT01760863), with Yang et al.'s study retained for its more recent data. |

***J Yang, J Parvizi, EN Hansen, et al.** 2020 Mark Coventry Award: Microorganism-directed oral antibiotics reduce the rate of failure due to further infection after two-stage revision hip or knee arthroplasty for chronic infection: a multicentre randomized controlled trial at a minimum of two years. *Bone Joint J* 2020;102-b:3-9.
