# Supplementary material for: The effect of extended postoperative oral antibiotic prophylaxis on the reinfection risk following two-stage exchange arthroplasty for hip and knee periprosthetic joint infection: a systematic review and meta-analysis
Source: BMC Musculoskelet Disord. 2026 Jan 8;27:101. doi: 10.1186/s12891-025-09410-4 (PMC12870892; doi:10.1186/s12891-025-09410-4)
Supplement: Supplementary file 2 — Supplementary Material 2. [file 12891_2025_9410_MOESM2_ESM.docx]

**Additional file 2**

**Additional file 2.** Details of data extraction from included studies.

| **Author/Year** | **Details of data extraction from included studies** |
| --- | --- |
| Zywiel 2011 | 1. The number of knees receiving oral antibiotics and those that did not, as well as the duration of oral antibiotic therapy following reimplantation, were obtained from the first paragraph of the Methods section. 2. The type of oral antibiotic was referenced in Figure 1. 3. The number of reinfections among patients treated with oral antibiotics and those who were not was obtained from the first paragraph of the Results section. |
| Johnson 2013 | 1. The number of hips treated with oral antibiotics and those not treated, and the duration of oral antibiotic therapy, were obtained from the fourth paragraph of the Methods section. 2. The number of reinfections in both groups was obtained from the first paragraph of the Results section. 3. The number of complications related to oral antibiotic use was obtained from the first paragraph of the Results section. |
| Yang 2020 | 1. The number of hips and knees that received or did not receive oral antibiotics, the number of reinfections in both groups, and the number of the specific joints affected by reinfection were obtained from Table I and Table III, following an intention-to-treat analysis. 2. The duration of oral antibiotic therapy was obtained from the second paragraph of the Methods section. 3. The side effects of oral antibiotics and the number of patients who discontinued oral antibiotic therapy due to these side effects were obtained from Table II and the second paragraph of the Results section. The number of reinfections caused by the same pathogen with identical antibiotic sensitivity was obtained from Table III. |
| Kelly 2022 | 1. The number of hips and knees that received or did not receive oral antibiotics, the number of reinfections in both groups, were derived from Tables 1 and 3. 2. The duration and type of oral antibiotics were from the first paragraph of the Results section. 3. The number of specific joints affected by reinfection were calculated from Table 4. 4. The number of reinfections caused by the same pathogen with identical antibiotic sensitivity was obtained from Table 4 and the last paragraph of the Results section. |
| Ryan 2023 | 1. The number of hips and knees was obtained from Table 2. 2. The number of specific joints affected by reinfection and the number of reinfections in three groups (> 2weeks, ≤ 2 weeks, and no oral antibiotic) were obtained from Table 3 and 4. 3. The duration of oral antibiotic therapy was obtained from the second paragraph of the “Materials and Methods” section. 4. The number of reinfections caused by the same pathogen with identical antibiotic sensitivity was from Table 5. |
